# Epidemiological Changes in Leishmaniasis in Spain According to Hospitalization-Based Records, 1997–2011: Raising Awareness towards Leishmaniasis in Non-HIV Patients

**DOI:** 10.1371/journal.pntd.0003594

**Published:** 2015-03-10

**Authors:** Zaida Herrador, Alin Gherasim, B. Carolina Jimenez, Marisol Granados, Juan Victor San Martín, Pilar Aparicio

**Affiliations:** 1 National Centre for Tropical Medicine, Health Institute Carlos III (ISCIII in Spanish), Madrid, Spain; 2 Network Biomedical Research on Tropical Diseases (RICET in Spanish), Madrid, Spain; 3 Médecins Sans Frontières, Madrid, Spain; 4 Department of Preventive Medicine, University Hospital Rio Ortega, Valladolid, Spain; 5 Department of Internal Medicine, University Hospital of Fuenlabrada, Madrid, Spain; Hospital Universitário Professor Edgard Santos, BRAZIL

## Abstract

In Spain, *Leishmania infantum* is endemic, human visceral and cutaneous leishmaniasis cases occurring both in the Peninsula, as well as in the Balearic Islands. We aimed to describe the clinical characteristics of leishmaniasis patients and the changes in the disease evolution after the introduction of antiretroviral therapy in 1997. In this descriptive study, we used Spanish Centralized Hospital Discharge Database for the hospitalized leishmaniasis cases between 1997 and 2011. We included in the analysis only the records having leishmaniasis as the first registered diagnosis and calculated the hospitalization rates. Disease trend was described taking into account the HIV status. Adjusted odds-ratio was used to estimate the association between clinical and socio-demographic factors and HIV co-infection. Of the total 8010 Leishmaniasis hospitalizations records, 3442 had leishmaniasis as first diagnosis; 2545/3442 (75.6%) were males and 2240/3442 (65.1%) aged between 14-65 years. Regarding disease forms, 2844/3442 (82.6%) of hospitalizations were due to visceral leishmaniasis (VL), while 118/3442 (3.4%) hospitalizations were cutaneous leishmaniasis (CL). Overall, 1737/2844 of VL (61.1%) were HIV negatives. An overall increasing trend was observed for the records with leishmaniasis as first diagnosis (p=0.113). Non-HIV leishmaniasis increased during this time period (p=0.021) while leishmaniasis-HIV co-infection hospitalization revealed a slight descending trend (p=0.717). Leishmaniasis-HIV co-infection was significantly associated with male sex (aOR=1.6; 95% CI: 1.25-2.04), 16-64 years age group (aOR=17.4; 95%CI: 2.1-143.3), visceral leishmaniasis aOR=6.1 (95%CI: 3.27-11.28) and solid neoplasms 4.5 (95% CI: 1.65-12.04). The absence of HIV co-infection was associated with lymph/hematopoietic neoplasms (aOR=0.3; 95%CI:0.14-0.57), other immunodeficiency (aOR=0.04; 95% CI:0.01-0.32) and transplant (aOR=0.01; 95%CI:0.00-0.07). Our findings suggest a significant increase of hospitalization in the absence of HIV co-infection, with a predomination of VL. We consider that clinicians in Spain should be aware of leishmaniasis not only in the HIV population but also in non HIV patients, especially for those having immunosuppression as an associate condition.

## Author Summary

Leishmaniasis is one of the most important human neglected parasitic diseases worldwide. Although it is considered a disease of the developing world, it is currently spreading northwards, outbreaks are occurring in endemic areas, and foci of the disease are appearing in previously non endemic countries, not necessarily associated to HIV as it occurred in the past. In the Old World, the disease has two clinical forms, cutaneous and visceral leishmaniasis. While the cutaneous form is mostly asymptomatic, the visceral form implies various degrees of severity, also being historically associated and influenced by the HIV co-infection. Since 1997, antiretroviral therapy (ART) became available for HIV treatment, subsequently influencing the evolution and epidemiology of leishmaniasis. In this paper we use hospitalized leishmaniasis discharge data, in order to describe this disease´s geographical and temporal trends and its main epidemiological characteristics by patients´ HIV status since ART introduction, in Spain between 1997 and 201.

## Introduction

Leishmaniasis represents a complex of mammalian diseases caused by parasitic protozoans classified as *Leishmania* species and spread through the bite of the sand fly. Two major clinical forms are known: cutaneous leishmaniasis (CL), causing scars and eventually disfiguration, and systemic or visceral leishmaniasis (VL) that can lead to deadly complications if left untreated [1]. Worldwide, at least 20 *Leishmania* species are causing leishmaniasis. Most foci occur in the tropics or subtropics, and only zoonotic *L*. *infantum* is transmitted in both the Old and the New World. *L*. *infantum* is the causative agent of both the cutaneous and visceral forms of leishmaniasis in Southwest Europe [2].

This zoonotic-vector-borne disease has a proven complex transmission dynamic; the seasonality of the vector species directly affects the transmission of the disease while the length of the activity period and sand fly densities are influenced by the climate conditions that affect their life cycle [3]. Moreover, the impacts of the sex, breed, age, use and habitat of the host dog on the emergence of this disease have been also explored in several epidemiological surveys [4]. In Spain, leishmaniasis is endemic in most of the Iberian Peninsula and the Balearic Islands. Two vector species are circulating, *P*. *pernicious* and *P*. *ariasi* [5]. *Phlebotomus sergenti*, the main vector of Leishmania *tropica*, is also represented in Spain, whilst *L*. *tropica* is not an endemic species in this country [4]. The dog is the main domestic reservoir, although wild mammals have been found infected with *L*. *infantum* [6]. Occasionally, outbreaks might occur.

From the mid-1980s to the late 1990s, a progressive increase in the number of cases of VL was observed in Spain, mainly due to the HIV pandemic [7]. At that time, one of the major challenges in controlling VL was its interaction with HIV infection, which increased the risk of developing active VL by 100 to 2320 times [1]. Most of the Leishmania/HIV co-infected cases occurred in medium age adult males, the disease presented mainly as VL [8]. These figures were obtained at a time when only a handful of weak HIV drugs existed. Since the introduction of highly active antiretroviral therapy (HAART) in January 1997, the number of Leishmania/HIV co-infections decreased to less than 10 cases in 2008 [7], also lowering the number of relapses and associated mortality [9]. This could be explained by the relatively good health condition of HIV patients treated with HAART but also because of an increased prescription of Amphotericin B, a treatment with less side effects than toxic antimonials [9]. However, VL and HIV co-infection has also been characterized by significantly lower cure rates, higher rates of drug toxicity, and higher relapse and mortality rates when compared with HIV-negative VL patients [10].

Currently, there is no national leishmaniasis control strategy in Spain, although some vectorial and reservoir control measures are periodically implemented in several Autonomous communities [5]. There are two main sources of information on human leishmaniasis epidemiology in Spain: the Spanish Centralized Hospital Discharge Database (CMBD, in Spanish) and the National Epidemiological Surveillance Network (RENAVE, in Spanish). Both databases have been previously analyzed [5,9,11]. However, epidemiological changes in terms of leishmaniasis patients’ characteristics and temporal trend after the introduction of HAART have not been thoroughly assessed. Therefore, this study aims to describe the hospital admissions and patients’ characteristics related to leishmaniasis disease and to assess the impact that recent changes on HIV epidemiology might have had on the epidemiology of human leishmaniasis in Spain.

## Methods

A retrospective descriptive study was conducted using the CMBD, which is a national hospital admission database managed by the Ministry of Health and Social Policy that includes all hospitalizations and where diagnoses are coded according to the Spanish version of the International Classification of Diseases, Ninth Revision, Clinical Modification (ICD-9-CM) [12]. The CMBD registry includes encrypted patient administrative and clinical data. It receives notification from around 98% of the public hospitals in Spain. Compulsory health insurance covers an estimated 99.5% of the registered Spanish population, although persons not covered by health insurance can receive treatment in public hospitals. Since 2005, CMBD also has a gradual coverage from private hospitals [13].

All hospital discharges for leishmaniasis (ICD 9 CM 085.0–085.9; in any diagnostic position) reported during a 15-year period (January 1, 1997 through December 31, 2011) were collected. A hospitalization was defined as each discharge medical record entered in the CMBD. For each entry, the following data were collected: sex, date of birth, autonomous community of residence, admission date, type of discharge, length of stay, diagnosis and health conditions other than leishmaniasis, surgical intervention during the stay and socioeconomic variables (institutionalization and lack of resources). Age was categorized in three groups: 0–15, 16–64 and ≥ 65 years old. These three age categories were selected to provide an overview of children, the working population and seniors. Days of stay in the hospital was categorized in ≤ 15 and > 16 days.

The diagnosis and health conditions collected were other related factors and underlying conditions for symptomatic leishmaniasis. These conditions were assessed by a search of all those codes associated with leishmaniasis occurrence and included: neoplasia, immunodeficiency other than HIV, transplantation and transplant failure, aplastic anemia, diabetes mellitus, hepatitis and long term use of steroids or other drugs (see S1 Text). ICD-9-CM codes 042 (HIV type 1), 079.53 (HIV type 2), V08 (asymptomatic HIV), and 795.71 (HIV nonspecific serological evidence) corresponding to HIV infection, in any diagnostic position, were computed as HIV-positive for analysis purposes.

The average number of hospitalizations per year and annual hospital admissions rate (per 1 million population) were calculated. Population at risk was obtained from the Spanish census projection [14]. It was assumed that the age distribution of the population covered by these hospitals was similar to the general population. Hospitalization rates were computed by region and year in order to assess temporal and geographical patterns. Results in terms of median rates were plotted in maps for the whole study period. Trends in hospitalization rates were analyzed by linear regression.

Differences in proportions were assessed by the χ2 test and 95% confidence intervals (95% CI) were calculated. Where a cell value was below 5, Fisher’s exact test for two-way tables was applied. Student's t test was used to compare differences in means. We used two-sided tests and p < 0.05 was considered significant.

Risk factors for leishmaniasis hospitalization were explored and stratified by HIV diagnosis, using bivariate analysis. Independent variables significantly associated with leishmaniasis hospitalization (p<0.1) were included in a forward stepwise multiple logistic regression model. Adjusted odds-ratio (aOR) and 95% confidence intervals were estimated. Where cell values were 0, 1 was added to each cell. The major assumptions of logistic regression analysis (absence of multicollinearity and interaction among independent variables) were checked to be satisfied. The goodness of fit of logistic regression models was assessed using Hosmer-Lemeshow statistic. We used PASW version 18.0 (SPSS Inc., Chicago, IL) and Arcgis version 10.0 software for data analysis.

### Ethics Statement

This study involves use of patient medical data from The Spanish National Hospital Database (CMBD). These data are hosted by the Ministry of Health Social Services and Equality (MSSSI). Researchers working in public and private institutions can request the databases by filling, signing and sending a questionnaire available at the MSSSI website. In this questionnaire a signed Confidentiality Commitment is required. All data is anonymized and de-identified by the MSSII before it is provided to solicitants.

## Results

### Spatial and temporal trends in Spain

A total of 8010 hospital discharges with diagnosis of leishmaniasis in any position were identified for the 15-year study period. Leishmaniasis diagnosis alone (without other diagnoses) occurred in 538 hospitalizations (6.7%), out of which 422 (78.4%), 24 (4.5%) and 92 (17.1%) were VL, CL and non-defined related forms, respectively. In 3442/8010 (42.8%) hospitalizations, leishmaniasis was registered as first diagnosis and further considered for the analysis; 2545/3442 (75.6%) were males, 2240/3442 (65.1%) belonged to the 14–65 age group.

Regarding the clinical form of leishmaniasis, 2844/3442 (82.6%) of hospitalizations were due to visceral leishmaniasis (VL), only 118/3442 (3.4%) hospitalizations were cutaneous leishmaniasis (CL), while 480/3442 (14.3%) were admitted as undetermined forms. For the whole database, which included leishmaniasis diagnosis in any position, similar figures were obtained: 6447/8010 (80.5%) VL, 225/8010 (2.8%) CL and 1338/8010 (16.7%) undetermined form. The distribution according to the age was slightly different, with a higher proportion of the 16–64 age group for the whole database than for those hospitalizations with leishmaniasis as first diagnosis. The percentage of HIV positive increased from 36.5% to 63.4% when we asses only those hospitalizations with leishmaniasis as first diagnosis and the whole database, respectively (Table 1).

**Table 1:** Descriptive analysis of leishmaniasis related hospitalizations, 1997–2011, Spain.

Variable		Related hospitalizations (n = 8010)		Leishmania as first diagnosis (n = 3442)	
		N	%	N	%
**Sociodemographic characteristics**					
**Sex**	**Female**	1653	20.6	897	26.1
	**Male**	6357	79.4	2545	75.6
**Age group**	**0–15**	930	11.6	820	23.8
	**16–64**	6461	80.7	2240	65.1
	**≥65**	619	7.7	382	11.1
**Clinical characteristics**					
**Clinical form of leishmaniasis**	**Cutaneous**	225	2.8	118	3.4
	**Visceral**	6447	80.5	2844	82.6
	**Non-defined**	1338	16.7	480	13.9
**HIV status**	**Positive**	5079	63.40	1255	36.46
	**Negative**	2931	36.60	2187	63.54

**Fig 1:**
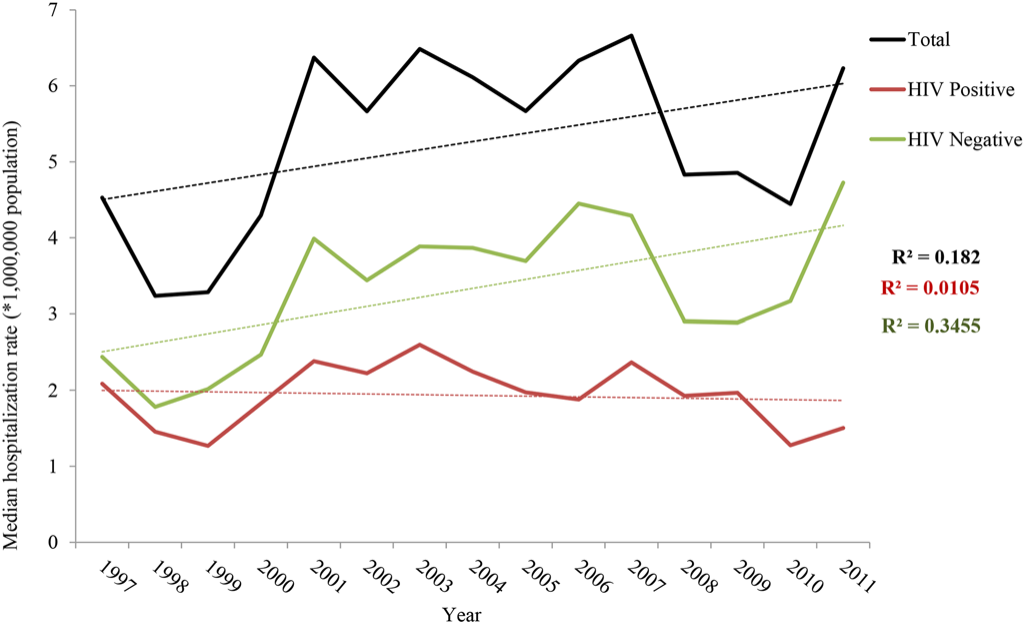
Temporal trend of hospitalizations with leishmaniasis as first diagnosis rates by HIV status, 1997–2011, Spain.

The temporal distribution of hospitalizations related to leishmaniasis as first diagnosis during the 15-year study period is represented in Fig. 1. At the national level, the median annual hospitalization rate was 5.6/1 million population (range 3.2–6.6/1 million population). The median annual hospitalization rate for hospitalized leishmaniasis-HIV co-infection was 1.9 (range 1.5 to 2.5), while for leishmaniasis hospitalizations without an HIV diagnosis was 3.4/1 million population (range 1.7 to 4.7). From 1997 to 2011, an increasing trend in the rates of hospitalizations with leishmaniasis as first diagnosis was observed (p = 0.113). Non-HIV leishmaniasis has also increased during this time period (p = 0.021) while leishmaniasis-HIV co-infection hospitalization revealed a slight descending trend (p = 0.717). These trends were similar to those observed when assessing all leishmaniasis related hospitalizations (n = 8010), with exception to the trend for the total population (regardless the HIV status), which shown a slightly descending trend (S1 Fig).

**Fig 2:**
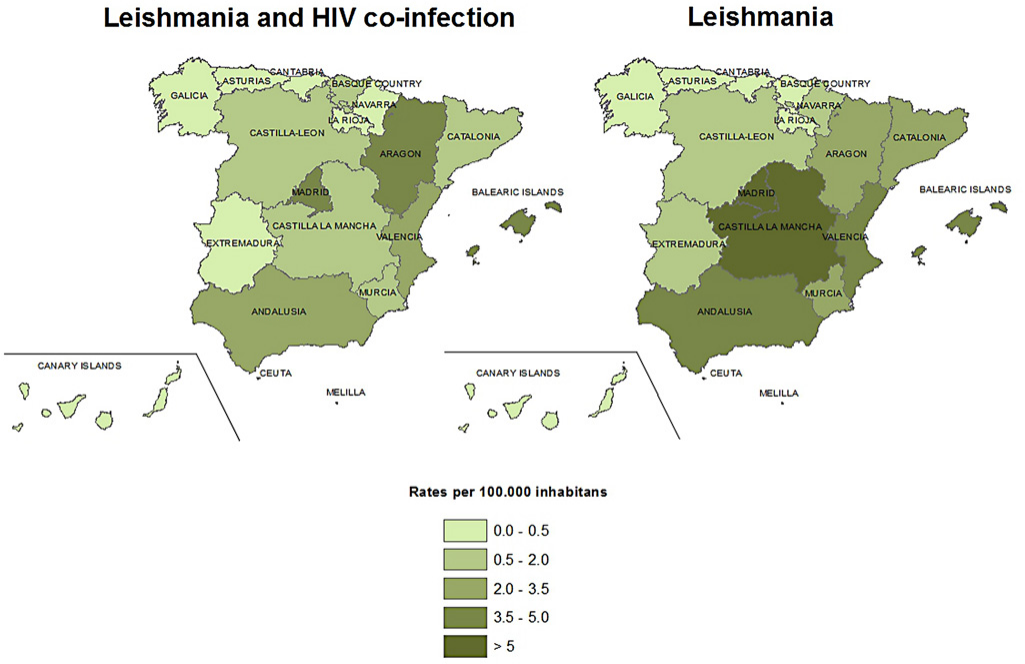
Median rates of hospitalizations with leishmaniasis as first diagnosis for HIV and non HIV leishmaniasis by autonomous community, Spain, 1997–2011.

Regarding the distribution of leishmaniasis hospitalizations (as first diagnosis) throughout the whole study period, the Madrid region had the highest median hospitalization rate (10.3 hospitalizations/1 million population), followed by the Balearic Islands (7.2/1 million population), Valencia (7.4/1 million population), Aragon (6.8/1 million population) and Castilla La Mancha (6.4/1 million population). The Northern autonomous communities of Spain (Cantabria, Navarra, Basque Country and Galicia) were among the ones with lower leishmaniasis hospitalization rates (S1 Table). The highest differences between leishmaniasis alone and leishmaniasis-HIV co-infection were registered in Castilla La Mancha, Catalonia and Murcia. Interestingly, Aragon was the only region where leishmaniasis-HIV co-infection hospitalization rate was higher than leishmaniasis alone (Fig. 2).

**Fig 3:**
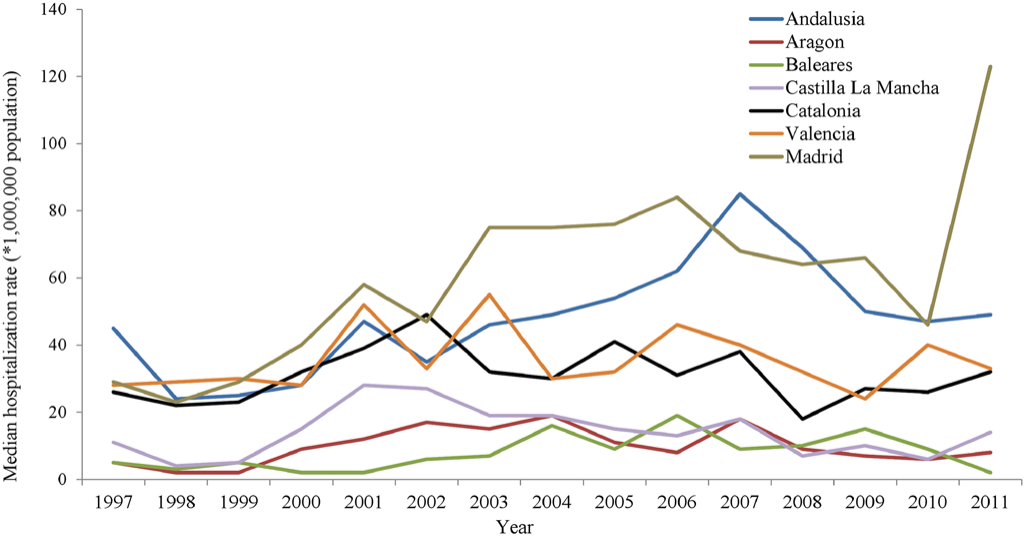
Median rates of Leishmaniasis hospitalizations with leishmaniasis as first diagnosis by autonomous community and year, in those communities with up to 100 cases.

The hospitalization trends within the regions with higher rates revealed different patterns. In Castilla La Mancha, hospitalization rates peaked in 2001 and were afterwards followed by a steady decline. The same decreasing trend was observed in Valencia. In the Balearic Islands, the hospitalization rate periodically reached high values during 2004, 2006 and 2009. A similar trend was identified in Aragon. In Madrid, after a significant increase (p = 0.01), a pick was reached towards the end of the study period, in 2011. The same tendency was identified in Andalusia (p = 0.05) while the Basque Country was the only region with a significant decrease in hospitalization rate (p = 0.042). The rest of the regions did not show significant tendencies (Fig. 3).

### Difference between leishmaniasis and leishmaniasis-HIV co-infection cases

In all leishmaniasis as first diagnosis hospitalization cases, information on HIV status was available. Of them, 1255/3442 (36.4%) were HIV positive. The proportion of males was higher among HIV positives compared to HIV negatives, 1084/1255 (86.4%) and 1461/2187 (66%), respectively. Nearly all HIV positives belonged to the 16–64 age group, 1248/1255 (99.4%), compared to 992/2187 (45.4%) of HIV negatives (Table 2).

**Table 2:** Differences among leishmaniasis and co-infected leishmaniasis-HIV cases based hospitalizations, with leishmaniasis as first diagnosis. Spain 1997–2011

Variables		Leishmania as first diagnosis (n = 3442)					
		VIH positive		VIH negative		Unadjusted OR	Adjusted OR
		N	%	N	%		
**No. of subjects**		1255	36.46	2187	63,54		
**Sex**	**Female**	171	13.6	726	33.2	1	1
	**Male**	1084	86.4	1461	66.8	2.22 (1.93–2.56)**	1.50 (1.17–1.91) *
**Age group**	**0–15**	0	0.0	820	37.5	1	1
	**16–64**	1248	99.4	992	45.4	1032 (144.9–7344) **	990 (138.76–7065.63) **
	**>65**	7	0.6	375	17.1	0.07 (0.01–0.53) **	0.02 (0.01–0.03) **
**Leishmaniasis**	**Cutaneous**	13	1.0	105	4.8	1	1
	**Visceral**	1107	88.2	1737	79.4	5.15 (2.88–9.20) **	6.07 (3.27–11.28) **
	**Non-defined**	135	10.8	345	15.8	0.32 (0.18–0.60) **	0.06 (0.06–0.77) **
**Type of discharge**	**No Exitus**	1252	94.3	2042	96.5	1	1
	**Exitus**	48	3.6	59	2.8	1.33 (0.90–1.96)	–
	**Unknown**	27	2.0	14	0.7	3.14 (1.64–6.02) **	4.18 (1.50–11.66) *
**Re-hospitalization**	**No**	887	70.7	1732	79.2	1	1
	**Yes**	368	29.3	455	20.8	1.56 (1.35–1.85) **	1.69 (1.35–2.12) **
**Surgery**	**No**	1185	94.4	1994	91.2	1	1
	**Yes**	70	5.6	193	8.8	0.61 (0.46–0.81) **	1.75 (1.24–2.47) **
**Days of stay**	**≤15 days**	852	67.9	1486	67.9	1	–
	**> 16 days**	403	32.1	701	32.1	1 (0.86–1.16)	–
**Solid neoplasm**	**No**	1230	98.0	2165	99.0	1	1
	**Yes**	25	2.0	22	1.0	2 (1.12–3.56)*	4.45 (1.65–12.04) *
**Lymph- hematopoietic neoplasms**	**No**	1246	99.3	2132	97.5	1	1
	**Yes**	9	0.7	55	2.5	0.28 (0.14–0.57) **	0.25 (0.11–0.57) *
**Other immunodeficiencies**	**No**	1255	99.9	2149	98.3	1	1
	**Yes**	1	1.0	37	1.7	0.05 (0.01–0.34) **	0.04 (0.01–0.32) *
**Transplant**	**No**	1255	100.0	2098	95.9	1	1
	**Yes**	0	0.0	89	4.1	0.01 (0.01–0.14) **	0.01 (0.00–0.07) **
**Transplant failure**	**No**	1245	99.2	2160	98.8	1	–
	**Yes**	10	0.8	27	1.2	0.64 (0.31–1.33)	–
**Aplastic Anemia**	**No**	1078	85.9	1856	84.9	1	–
	**Yes**	177	14.1	331	15.1	0.92 (0.76–1.12)	–
**Diabetes Mellitus**	**No**	1244	99	2009	91.9	1	1
	**Yes**	12	1	177	8.1	0.11 (0.06–0.20) **	0.10 (0.05–0.18) **
**Hepatitis**	**No**	1112	88.6	2153	98.4	1	1
	**Yes**	143	11.4	34	1.6	8.14 (5.56–11.92) **	3.95 (2.52–6.20) **
**Long term use of steroids**	**No**	1255	100.0	2171	99.3	1	1
	**Yes**	0	0.0	16	0.7	0.11 (0.01–0.81) *	0.69 (0.05–9.65)
**Long-term drug use**	**No**	1219	97.1	2168	99.1	1	1
	**Yes**	36	2.9	19	0.9	3.37 (1.92–5.19) **	2.97 (1.40–6.33) *
**Alcoholism**	**No**	1183	94.3	2143	98.0	1	1
	**Yes**	72	5.7	44	2.0	2.96 (2.02–4.34) **	1.76 (1.12–2.78) *
**Institutionalized**	**No**	1147	91.4	2169	99.2	1	1
	**Yes**	108	8.6	18	0.8	11.35 (6.85–18.78) **	3.96 (2.27–6.91) **
**Lack of resources**	**No**	1245	99.2	2183	99.8	1	1
	**Yes**	10	0.8	4	0.2	4.38 (1.37–14.03) *	4.53 (0.84–24.46)

* p<0.05;

** p<0.001.

Visceral leishmaniasis was the predominant clinical form both in HIV positives 1107/1255 (88.2%) and in HIV negatives 1737/2187 (79.4%). The most frequent co-morbidities documented in the HIV positive group were aplastic anemia 177/1255 (14.1%), hepatitis 143/1255 (11.4%) and alcoholism 72/1255 (5.7%). There was a history of surgery in 70/1255 (5.6%) and of institutionalization in 108/1255 (8.6%) of HIV positive records. In the HIV negative group, the most frequent comorbidities or conditions were aplastic anemia, history of surgery and diabetes with 331/2187 (15.1%), 193/2187 (8.8%) and 177/2187 (8.1%) records, respectively (Table 2).

Leishmaniasis hospitalization with HIV co-infection was significantly associated with male sex, (aOR = 1.6 (95% CI 1.25–2.04)), and age group 16–64 years (aOR = 17.4 (95%CI 2.1–143.3)). Regarding the disease outcome, patients hospitalized with HIV co-infection where 1.3 times more likely to decease, compared to hospitalized patients without HIV infection (95%CI 0.9–1.96). The hospitalized individuals with leishmaniasis HIV co-infection were also 6 times more likely to have visceral leishmaniasis compared with those without HIV infection, and 4 times more likely to have a solid neoplasm diagnosis. They were also 4 times more likely to have a positive hepatitis diagnosis. Institutionalization and a history of long term drug use were significantly associated with hospitalization with Leishmania HIV co-infection. The hospitalization period did not differ between HIV co-infected leishmaniasis hospitalizations and non HIV leishmaniasis hospitalizations, and predominantly lasted less than 2 weeks (Table 2).

## Discussion

This study provides a 15-year review of the epidemiological trends and patient characteristics of Leishmania hospitalizations in Spain. We particularly covered all the antiretroviral therapy era since 1997 and stratified the results by HIV status. The CMBD database provides a reliable picture of the Spanish leishmaniasis related hospitalization as it covers 98% of public hospital´s admissions. Public healthcare insurance is covering almost 100% of the Spanish population as of 2011, and private hospitals represent only a small proportion of all hospital admissions [15].

During this 15-year period, there were 8010 hospitalizations for which leishmaniasis codes were entered in any diagnostic position among which 3442 records had leishmaniasis codes in the first position. The majority of the overall hospitalizations belonged to the 16–64 age group and this could be regarded as within the general tendency of hospitalizations in Spain. On the other hand, within the hospitalizations due to leishmaniasis as first diagnosis, the higher percentage of the 0–15 years age group compared to the overall hospitalization group could be seen as due to the disease’s characteristics [5]. However, given the similarities between both groups for the other main characteristics and because readmissions could not be clearly identified with the database, we decided to consider for further analysis only the 3442 records were leishmaniasis was the first diagnosis. As in previous studies that have used the CMBD, we assumed that the first diagnosis was the cause of admission [5,9,11].

It is generally admitted that, worldwide, the true burden of leishmaniasis disease is underestimated [16]. In Spain, leishmaniasis was declared a notifiable disease from 1982 to 1995. Since 1996, a new decentralized surveillance system (RENAVE) was implemented and leishmaniasis became a notifiable disease in those regions where it is considered endemic (12 out of 17 autonomous communities: Andalucía, Aragón, Baleares, Cantabria, Castilla-León, Cataluña, Valencia, Extremadura, Madrid, Murcia, Navarra and La Rioja) [5]. Between 1996 and 2011, RENAVE detected 1755 leishmaniasis cases [5,9], almost half of CMBD records with leishmaniasis codes in first position (3442 cases from 1997 to 2011). The difference in reported cases between the two systems clearly suggests that underreporting is an important aspect in Spain with a non-negligible magnitude.

In our study, the geographical distribution shows that hospitalizations have been reported from all over the country. Previous epidemiological reviews have also detected cases in all peninsular regions of Spain and the Balearic islands as well as a homogenization of the distribution when considering the HIV co-infected cases [5,11]. Given this continued reporting from all regions, the non-endemic condition of some regions should be reconsidered as should be the attitudes towards strengthening the disease surveillance in these areas. According to RENAVE, the regions with the highest incidence during 1996–2011 were Baleares, Valencia, Madrid, Andalusia and Catalonia [5]. Although this geographical distribution seems similar to our figures, the picture changes when we stratified by HIV status, Castilla La Mancha and Aragon placing second in hospitalization rates for non-HIV and HIV co-infected cases, respectively. Several factors could be responsible for the differences in leishmaniasis hospitalization rates encountered between autonomous regions. The distribution of HIV cases and related risk factors within different autonomous regions could be playing a role in the differences on hospitalizations rates [17]. On the other hand, the epidemiological characteristics of Leishmania and HIV co-infection can differ from one autonomous community to another due to parasite strain and other differences in aetiological agent circulation (involving parasite, host and reservoir), vector behaviour [3] and, possibly, associated regional geographic and climatic factors or socio-environmental conditions of the exposed hosts [18].

We have found an increasing trend in leishmaniasis as first diagnosis hospitalization rates from 1997 to 2011, although this trend was statistically significant only for non-HIV cases. Moreover, the analysis of the temporal distribution of leishmaniasis hospitalization rates in Spain shows an endemic pattern with increases every 3–4 years, suggesting the existence of localized outbreaks. It is during these “peaks” when the differences in figures encountered by HIV-status seem greater. Looking at the temporal distribution by autonomous regions (Fig. 3), we can observe that this increasing trend occurs mainly at the expense of the community of Madrid, where one of the most important Leishmaniasis outbreaks in Europe, starting from 2010, has taken place. According to Arce *et al*., in this outbreak, only 4% of all leishmaniasis cases were coinfected with HIV [19]. The risk of Leishmania onset for HIV infected persons during a Leishmania outbreak is unclear and must probably depend on the HIV-status and adherence treatment. Further research is needed to clarify this issue.

Other trends and peaks, like those in Andalusia, also suggest the existence of outbreaks but there are no literature referrals describing such outbreaks. It would therefore be required to carry out retrospective analyses to better characterize these possible outbreaks using the data available at the national epidemiological network (RENAVE). This would provide valuable information and would help add new knowledge about the epidemiological picture of leishmaniasis in Spain.

Regarding the characteristics of the cases registered as Leishmaniasis as first diagnosis, patients were predominantly male, belonging to the age group 16–64 years and admitted for visceral leishmaniasis. This patient profile has constantly been identified in previous studies and the predominance of visceral leishmaniasis is related to the hospitalization-based data source [9,11]. HIV stratification slightly reduces the differences between sexes and age groups (Table 2). Thus, age group 0–14 accounts for more than 30% of records among HIV negatives while there were no records for this age group among HIV positives. However, males are still largely prevalent in HIV negatives, which seems to be concordant with findings from previous epidemiological studies indicating that VL occurs more frequently among adult males [20]. Although the role of sex hormones has been hypothesized in the modulation of immunity against leishmaniasis [21,22], the explanation for this trend still remains uncertain.

As can be observed in Table 2, stratification by HIV status proves to be a very helpful method to further characterize leishmaniasis hospitalizations. Significant differences were observed for most of the variables included in the analysis, notably solid neoplasm, hepatitis, long term drug use, alcoholism, institutionalization and lack of resources, all of them more likely to be present in HIV hospitalized patients. Interestingly, non HIV hospitalized patients were more likely to present other conditions such as lymphohematopoietic neoplasms, immunodeficiency, transplantation, diabetes mellitus and long term use of steroids. It is well-known that malnutrition and immunosuppression may reactivate latent Leishmania infection, thus becoming risk factors particularly for the visceral form [23]. However most research has been focused in acquired immunodeficiency [21].

In HIV co-infected cases, the literature shows that cure trends are lower and relapse rates and mortality are higher, when compared to non HIV leishmaniasis patients [10]. In our study, HIV co-infection was not significantly associated with higher mortality. This supports findings in previous studies where no difference in mortality could be found in the HIV leishmaniasis co-infected patients group compared to leishmaniasis without HIV [9]. However, another similar paper revealed a significant association between HIV co-infection and higher mortality [11]. It is also interesting to highlight the fact that the average hospitalization length was under 2 weeks in two thirds of hospitalizations in both HIV and non HIV cases. This could be explained by an extended use of amphotericin B reflecting the efficacy of the drug together with the reduced toxicity [9]. On the contrary, the high and significant OR found for the two variables analysing socioeconomic factors suggest that these still play a major role in the HIV population vulnerability to leishmaniasis. Further investigation is needed in order to confirm or dismiss this association in the highly active antiretroviral therapy era.

### Limitations and conclusions

The CMBD does not provide information about parasite isolation, detection, PCR or serologic test for diagnosis of visceral leishmaniasis (VL). However, the CMBD database provides reliable information to support decision-making based on ICD-9 codification carried out by medical doctors. It has been proved to be a trusted source for many epidemiological studies [9,11,24]. Leishmaniasis is endemic in Spain. Diagnosis is not performed on primary health care level, but in hospitals [25]. Human leishmaniasis diagnosis fits the case definition criteria given by WHO: “a case of visceral leishmaniasis is a person showing clinical signs (mainly prolonged irregular fever, splenomegaly and weight loss), with serological and/or parasitological confirmation” [1]. As common practice, in Spanish hospitals the diagnosis of VL is mainly based on the bone marrow aspirate, either by direct microscopy visualization or PCR detection [25]. CMBD database does not reveal the exact number of patients who have been diagnosed with other techniques, such as serology, but it is probably small.

The CMBD provides a complete record of all hospitalizations and is not subject to the limitations of outpatient surveillance systems. Still, the CMBD does not show the real incidence of Leishmania infection, as cutaneous infections are mainly treated in primary care centers and not entered in this registry [9]. Another limitation of the CMBD is that data is anonymous so it is not possible to confirm if the same patient was admitted more than once in different calendar years. Furthermore, it is not possible to identify admission of the same patient to different hospitals.

Since highly active antiretroviral therapy was introduced in 1997, a decrease in the number of leishmaniasis cases might have been expected. However, a significant decrease has only been reported for co-infected cases [10]. On the other hand, leishmaniasis is currently spreading northwards in endemic regions, outbreaks are occurring in endemic areas, and foci of the disease are appearing in previously non endemic European countries [2,10]. Similarly to previous papers [9,11], we have observed a change in the epidemiology of VL hospitalizations, with the increase of non-HIV VL cases. This can have direct clinical implications. Classically, VL in Spain has been associated to the HIV epidemic and clinicians regularly consider this diagnosis in HIV patients presenting compatible clinical signs and symptoms [7,22,26]. However, the findings of the present study show a changing clinical pattern with other co-morbidities. This requires a special clinical consideration for several reasons; for example, the increased numbers of subjects receiving therapy for autoimmune diseases, cancer or transplants that result in suppressed cellular immunity [10,23], as is the disease burden of diabetes mellitus type 2 [27] and other prevalent co-morbidities in the Spanish population [28], probably playing a role in the progression to symptomatic leishmaniasis. Moreover, there is limited awareness of the disease among clinicians other than those treating infectious diseases. In conclusion, we consider that clinicians in Spain should be aware of leishmaniasis not only in the HIV population but also in non HIV patients and particularly in those presenting the above referred comorbidities.

## Supporting Information

S1 Text ICD-9 codes for underlying conditions and related factors for leishmaniasis disease, 1997–2011, Spain. (XLSX)

S1 Table Leishmaniasis hospitalization rates by HIV status and autonomous region, 1997–2011, Spain. (XLSX)

S1 Fig Temporal trend of leishmaniasis hospitalizations (at any diagnostic position) rates by HIV status, 1997–2011, Spain. (TIF)

S1 Checklist STROBE Checklist. (DOC)
