# Correction: Epidemiological Changes in Leishmaniasis in Spain According to Hospitalization-Based Records, 1997-2011: Raising Awareness towards Leishmaniasis in Non-HIV Patients

**DOI:** 10.1371/journal.pntd.0004379

**Published:** 2016-01-15

**Authors:** Zaida Herrador, Alin Gherasim, B. Carolina Jimenez, Maria del sol Granados, Juan Victor San Martín, Pilar Aparicio

The fourth author’s name is spelled incorrectly. The correct name is Maria del sol Granados. The correct citation is: Herrador Z, Gherasim A, Jimenez BC, Granados M, San Martín JV, Aparicio P (2015) Epidemiological Changes in Leishmaniasis in Spain According to Hospitalization-Based Records, 1997–2011: Raising Awareness towards Leishmaniasis in Non-HIV Patients. PLoS Negl Trop Dis 9(3): e0003594. doi:10.1371/journal.pntd.0003594
