# Supplementary figures and images for: Epidemiological Changes in Leishmaniasis in Spain According to Hospitalization-Based Records, 1997–2011: Raising Awareness towards Leishmaniasis in Non-HIV Patients
Source: PLoS Negl Trop Dis. 2015 Mar 10;9(3):e0003594. doi: 10.1371/journal.pntd.0003594 (PMC4355586; doi:10.1371/journal.pntd.0003594)

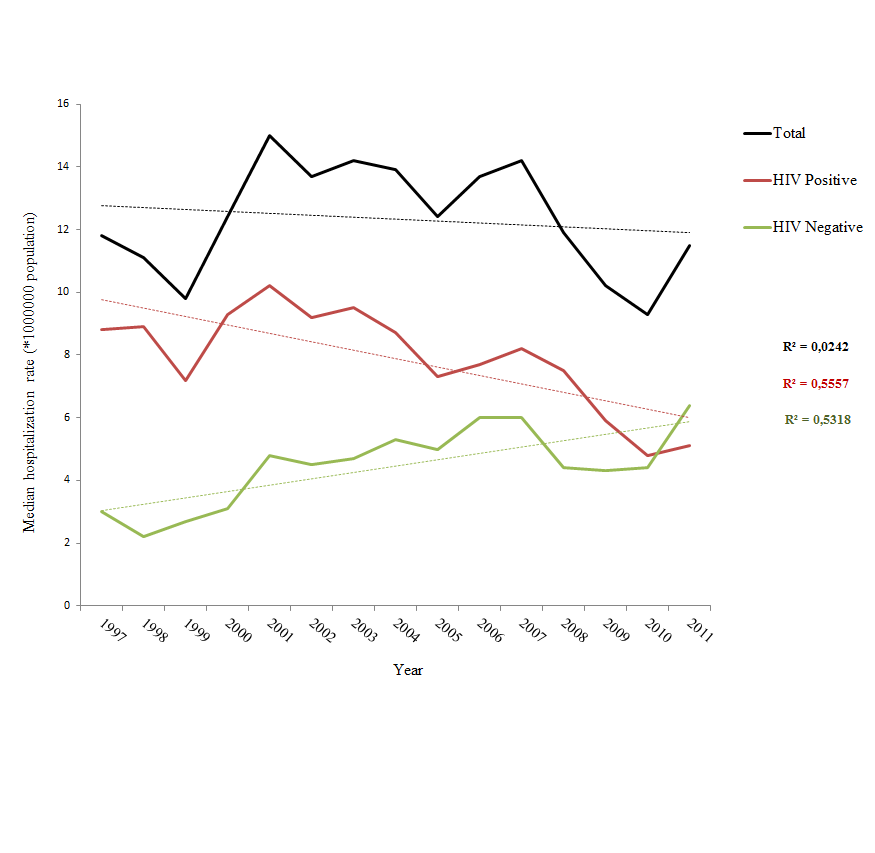

Supplement: S1 Fig — (TIF) [file pntd.0003594.s003.tif]
